# Superior Thyroid Artery of MLS-preserved Cadavers: A New Microsurgery Training Model

**DOI:** 10.4274/balkanmedj.galenos.2019.2019.8.47

**Published:** 2019-12-20

**Authors:** Mehmet Emre Yeğin, Servet Çelik, Okan Bilge

**Affiliations:** 1 Clinic of Plastic Surgery, Elazığ Fethi Sekin City Hospital, Elazığ, Turkey; 2 Department of Anatomy, Ege University School of Medicine, İzmir, Turkey

## To the Editor,

Microsurgery is a specialized area of surgery that demands training and research at every step. As a useful material for training, experimental methods are important for this purpose. However, ethical and economic considerations have forced scientists to seek alternative training materials. Therefore, in this study we aimed to use embalmed human ca-daver tissue, which are similar to live structures.

**Figure 1:**
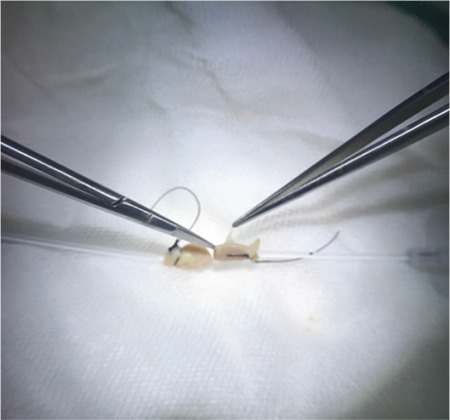
Adventisectomy of the vessel. Note that the flexibility and fragility of the adventitial tissue is just sufficient for dissec-tion and cutting.

**Figure 2:**
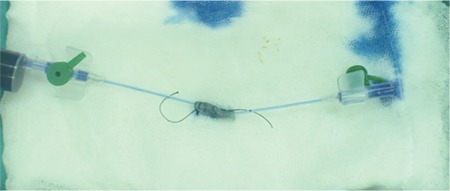
Pulsatile methylene blue injection mimics the blood flow in an anastomosed vessel.

The superior thyroid artery of a Modified Larssen Solution (MLS)-preserved cadaver was laid on saline-soaked gauze. Cannulas were fixed on both sides with silk sutures. One milliliter of saline was injected into the vessel lumen and allowed to drain from the other side. During this procedure, it was observed that the tissue caliber and turgor were suf-ficient to mimic the pulse. Subsequently, an incision was made in the middle of the artery. Adventisectomy was per-formed, and it was observed that the adventitia dissects and pierces identically to living tissues (Figure 1). Anastomo-sis was performed by a routine microsurgical technique using a 9/0 nylon suture. Patency was checked with a 1-mL injection of pulsatile methylene blue, and the evacuation of methylene blue from the other side was observed, mimick-ing pulsatile reactions of the vessel wall (Figure 2).

There is very limited information about the use of cadaver-embalming techniques in microsurgical education. One of the best representatives of this topic is a study by Wolff et al. (1), which used a technique called Thiel’s method. Their study shows similar visual quality and tissue properties to the ones mentioned here. In their study, they used a lateral circumflex femoral artery with a diameter to that of the superior thyroid artery (1).

Larssen Solution was first introduced by Sampaio and modified by Guimaraes Da Silva. (2) It was proposed as an al-ternative to the formaldehyde embalming technique because of its toxic side effects. It consists of a very low amount of formaldehyde compared with Thiel’s solution, which is another fixative solution with similar properties and is used widely (3). Moreover, the main disadvantage of Thiel’s technique is its higher cost compared with our technique ($70 to $330) (2).

MLS has been reported as a feasible technique for laparoscopic and instrumentation training (2). Another report ques-tioning the usability of MLS in plastic surgery training has been published (4). This pilot study shows that the superi-or thyroid artery of cadavers preserved in MLS is a good example of an accessible and cost-effective microsurgery train-ing method.
